# Transmission of survival signals through Delta-like 1 on activated CD4^+^ T cells

**DOI:** 10.1038/srep33692

**Published:** 2016-09-23

**Authors:** Takahiro Furukawa, Chieko Ishifune, Shin-ichi Tsukumo, Katsuto Hozumi, Yoichi Maekawa, Naoko Matsui, Ryuji Kaji, Koji Yasutomo

**Affiliations:** 1 Department of Immunology & Parasitology, Graduate School of Medicine, Tokushima University, Tokushima, Japan; 2 Department of Clinical Neuroscience, Graduate School of Medicine, Tokushima University, Tokushima, Japan; 3 Department of Immunology, Tokai University, Isezaki, Kanagawa, Japan; 4 Department of Parasitology and Infectious Diseases, Gifu University Graduate School of Medicine, Gifu, Japan

## Abstract

Notch expressed on CD4^+^ T cells transduces signals that mediate their effector functions and survival. Although Notch signaling is known to be cis-inhibited by Notch ligands expressed on the same cells, the role of Notch ligands on T cells remains unclear. In this report we demonstrate that the CD4^+^ T cell Notch ligand Dll1 transduces signals required for their survival. Co-transfer of CD4^+^ T cells from Dll1^−/−^ and control mice into recipient mice followed by immunization revealed a rapid decline of CD4^+^ T cells from Dll1^−/−^ mice compared with control cells. Dll1^−/−^ mice exhibited lower clinical scores of experimental autoimmune encephalitis than control mice. The expression of Notch target genes in CD4^+^ T cells from Dll1^−/−^ mice was not affected, suggesting that *Dll1* deficiency in T cells does not affect cis Notch signaling. Overexpression of the intracellular domain of Dll1 in *Dll1*-deficient CD4^+^ T cells partially rescued impaired survival. Our data demonstrate that Dll1 is an independent regulator of Notch-signaling important for the survival of activated CD4^+^ T cells, and provide new insight into the physiological roles of Notch ligands as well as a regulatory mechanism important for maintaining adaptive immune responses.

CD4^+^ T cells are central for controlling adaptive immune responses directed against pathogens by differentiating toward a variety of effector cells upon recognition of antigens presented by MHC class II^1^,^2^. Following the activation and proliferation of pathogen-specific CD4^+^ T cells, a fraction of the activated CD4^+^ T cells survive as long-lived memory CD4^+^ T cells that respond with enhanced kinetics and effector functions to subsequent infections^3^,^4^.

In mammals, there are four Notch receptors (Notch1-4) and five Notch ligands; Delta-like (Dll) 1, 3, and 4 and Jagged 1 and 2. Notch/Notch ligand interactions allow Notch receptors to be proteolytically cleaved in the extracellular domain by ADAM (a distintegrin and metalloprotease), which is followed by cleavage in the transmembrane domain by a γ-secretase complex. The intracellular domain of Notch translocates into the nucleus and interacts with Rbpj. We and other groups revealed that Notch signaling regulates a variety of aspects of mature T cell differentiation as well as intrathymic T cell development^5^,^6^,^7^,^8^,^9^,^10^,^11^. We also revealed that Notch signaling is essential for the survival of memory CD4^+^ T cell^12^, and others have reported that Notch protects activated CD4^+^ T cells from apoptosis^13^.

Similar to Notch receptors, Notch ligands also undergo ADAM-mediated cleavage in their extracellular domains followed by processing by γ-secretase^14^,^15^. The processing of Notch ligands downregulates Notch signaling in neighboring cells while Notch receptors interact with their ligands in the same cells. This *cis*-acting activity decreases Notch signaling^16^,^17^,^18^,^19^, although the detailed molecular mechanism for *cis*-inhibition remains unclear. Several reports have demonstrated that the intracellular domain of Notch ligands translocates into the nucleus and controls gene expression^20^,^21^. However, as those findings have not been repeated by *in vivo* studies, the physiological roles of signal transmission through Notch ligands remain unclear.

In this report we investigated the roles of Dll1 in mature CD4^+^ T cell function. We found that deletion of *Dll1,* but not *Jagged1*, in T cells disturbed the maintenance of activated/memory CD4^+^ T cells. The deletion of *Dll1* in CD4^+^ T cells attenuated the severity of experimental autoimmune encephalomyelitis (EAE) and was associated with impaired differentiation of Th1 and Th17 cells. These data identify a novel mechanism for Dll1-mediated maintenance of activated/memory CD4^+^ T cells.

## Results

### Normal T cell development and activation in the absence of Dll1 in T cells

**Figure 1:**
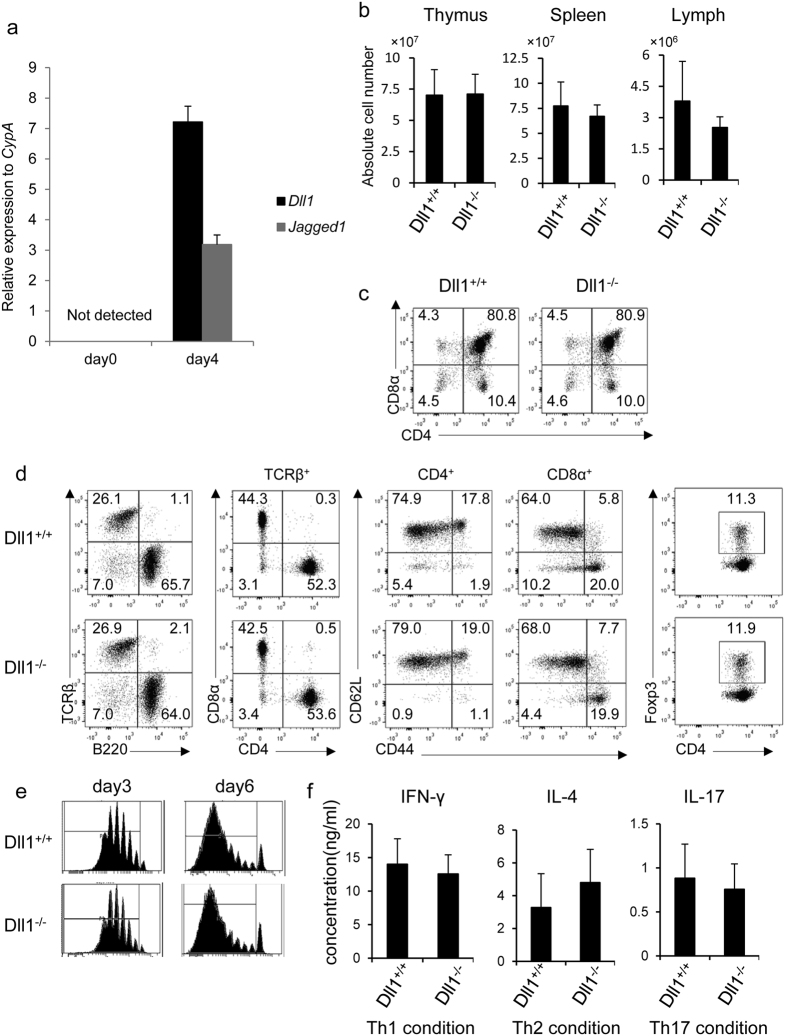
(**a**) Spleen cells from C57BL/6 mice (n = 5) were stimulated with anti-CD3 mAb (1 μg/ml) for 4 days. The expression of Dll1 or Jagged1 on naïve or activated CD4^+^ T cells was evaluated by real-time PCR. The data shown are mean ± S.D. (**b**) Spleen cells and thymocytes from Dll1^+/+^ or Dll1^−/−^ mice (n = 5) were counted and the mean ± SD is shown. (**c**) Thymocytes or (**d**) spleen cells were stained with the indicated antibodies and analyzed by flow cytometry. The numbers in the figures indicate the relative frequency of cells in each column. (**e**) Spleen cells from Dll1^+/+^ or Dll1^−/−^ mice were CFSE-labeled and stimulated with anti-CD3 mAb (1 μg/ml) for 3 or 6 days. CFSE dilution was measured by flow cytometry. (**f**) Spleen cells from Dll1^+/+^ or Dll1^−/−^ mice were stimulated with anti-CD3 mAb (1 μg/ml) under Th1, Th2, or Th17 conditions for 3 days. After purification of CD4^+^ T cells, CD4^+^ T cells were stimulated with plate-bound anti-CD3 and anti-CD28 mAb for 1 day. Supernatants were measured by ELISA after 1 day of culture. The data in these figures are representatives of four independent experiments.

We first examined the mRNA expression of Dll1 and Jagged1 on naïve and activated CD4^+^ T cells. Neither the mRNA of Dll1 nor Jagged1 was detected on naïve CD4^+^ T cells (Fig. 1a). In contrast, Dll1 and Jagged1 mRNA is upregulated in 4-day activated CD4^+^ T cells (Fig. 1a). Cell surface Dll1, but not Jagged1, was detected in activated CD4^+^ T cells by flow cytometry (Supplementary Figure 1). The expression of cell surface Dll1 was not detected on activated CD4^+^ T cells from *Dll1*^flox/flox^ X CD4-*Cre* transgenic (Dll1^−/−^) mice (Supplementary Figure 1). Therefore, we focused our studies on Dll1 on activated CD4^+^ T cells.

We first assessed the development of T cells in the thymus and spleen of Dll1^−/−^ and *Dll1*^+/+^ mice crossed with CD4-*Cre* transgenic (Dll1^+/+^) mice. The total cell number of thymocytes and spleen cells in Dll1^−/−^ mice was equivalent to that of Dll1^+/+^ mice (Fig. 1b). The frequency of CD4^+^CD8^+^, CD4^+^CD8^−^ or CD4^−^CD8^+^ cells in the thymus was comparable between Dll1^−/−^ and Dll1^+/+^ mice (Fig. 1c). The frequency of TCRβ^+^, CD4^+^TCRβ^+^ or CD8^+^TCRβ^+^ cells, the expression pattern of CD44 and CD62L in CD4^+^ and CD8^+^ cells, and the expression of Foxp3 in CD4^+^ cells in the spleen of Dll1^−/−^ mice were equivalent to those of Dll1^+/+^ mice (Fig. 1d). These data suggest that *Dll1* deficiency in T cells does not affect T cell development in the thymus and spleen.

We next sought to determine if deletion of *Dll1* in T cells affects CD4^+^ T cell proliferation or functional differentiation. Purified splenic CD4^+^ T cells from Dll1^−/−^ or Dll1^+/+^ mice were labeled with CFSE and stimulated with anti-CD3 and anti-CD28 antibodies for 3 or 6 days. Cell division as evaluated by CFSE dilution was comparable between the two types of cells at both 3 and 6 days post-stimulation (Fig. 1e). Spleen cells from Dll1^−/−^ mice were stimulated with anti-CD3 mAb under Th1 (IL-12 and anti-IL-4 mAb), Th2 (IL-4 and anti-IFN-γ mAb) or Th17 (IL-6, TGF-β, IFN-γ, IL-4) conditions and IFN-γ, IL-4 or IL-17, respectively, were measured by ELISA after 3 days of stimulation. The concentration of each cytokine for the three culture conditions was comparable between the two types of cells (Fig. 1f). Taken together, these data demonstrate that *Dll1* deficiency in T cells does not affect T cell development or the functional differentiation of CD4^+^ T cells.

### The survival of activated CD4^+^ T cells in Dll1^−/−^ mice is impaired

**Figure 2:**
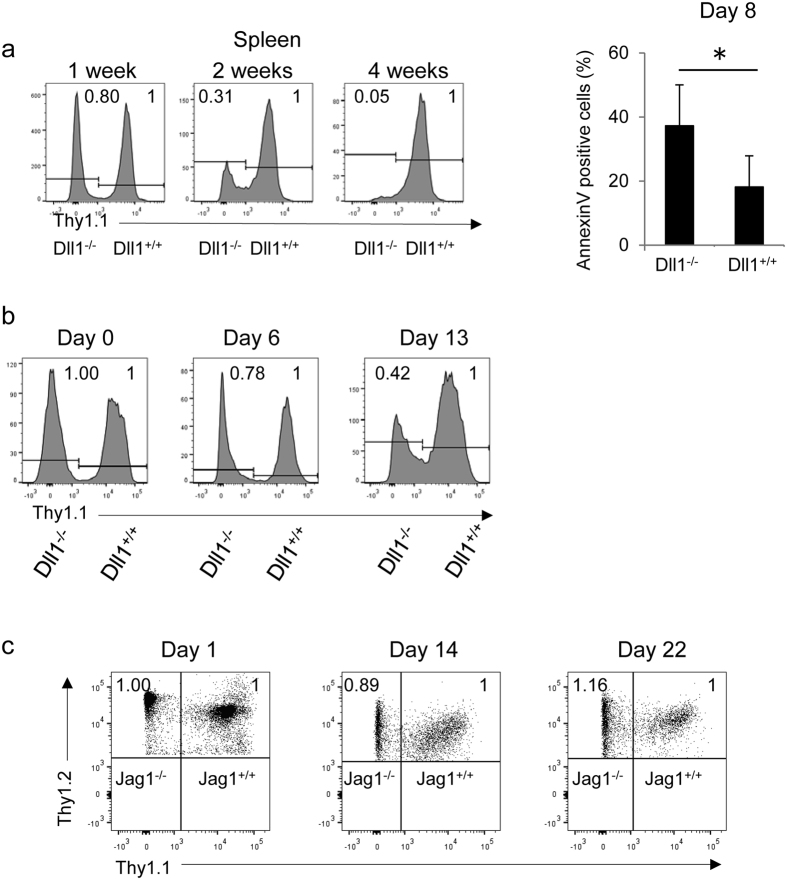
(**a**) Identical numbers of T cells from Dll1^−/−^: OT-II (Thy1.2^+^) or Dll1^+/+^: OT-II (Thy1.1^+^) mice were transferred into unirradiated C57BL/6 (Thy1.1^+^Thy1.2^+^) mice (n = 8) that were subsequently immunized by OVA in CFA. The relative cell numbers of Thy1.1^−^Thy1.2^+^ or Thy1.1^+^Thy1.2^−^ cells were evaluated by flow cytometry. The Annexin V^+^ cells in Thy1.1^−^Thy1.2^+^ or Thy1.1^+^Thy1.2^−^ cells were evaluated by flow cytometry 8 days after immunization. The data shown are mean ± S.D. *Indicates a statistical difference (*p* < 0.05). (**b**) Purified CD4^+^ T cells from Dll1^−/−^: OT-II (Thy1.1^−^Thy1.2^+^) or Dll1^+/+^: OT-II (Thy1.1^+^ Thy1.2^+^) mice were stimulated with OVA protein (50 μg/ml) with irradiated spleen cells from C57BL/6 mice (Thy1.1^+^ Thy1.2^−^) and the relative cell numbers of Thy1.1^−^ and Thy1.1^+^ cells gated on Thy1.2^+^ cells were evaluated by flow cytometry at the indicated times. (**c**) Identical numbers of T cells from Jagged1^−/−^: OT-II (Thy1.2^+^) or Jagged1^+/+^: OT-II (Thy1.1^+^) mice were transferred into unirradiated C57BL/6 (CD45.1) mice (n = 8) that were subsequently immunized with OVA in CFA. The relative cell number of Thy1.1^−^Thy1.2^+^ or Thy1.1^+^Thy1.2^−^ cells was evaluated by flow cytometry. The data in these figures are representative of three independent experiments.

In order to examine the roles of Dll1 in the survival of activated CD4^+^ T cells, we crossed Dll1^−/−^ mice with OT-II TCR transgenic (Dll1^−/−^: OT-II) mice (Thy1.1^−^Thy1.2^+^). The same number of CD4^+^ cells from Dll1^−/−^: OT-II mice and those from control Dll1^+/+^: OT-II mice (Thy1.1^+^Thy1.2^+^) were transferred into C57BL/6 mice (Thy1.1^+^Thy1.2^−^) that were subsequently immunized with OVA protein emulsified in CFA. The relative cell number of Dll1^−/−^: OT-II gradually decreased, and cells from Dll1^−/−^: OT-II mice were nearly absent 4 weeks after immunization (Fig. 2a). The total cell number was also decreased in Dll1^−/−^: OT-II compared with control cells 16 days after immunization (Supplementary Figure 2). In accordance with the rapid decline of CD4^+^ T cells from Dll1^−/−^: OT-II mice, Annexin V^+^ cells were increased in Dll1^−/−^ mice compared with those from Dll1^+/+^: OT-II mice 8 days after immunization (Fig. 2a). We also compared T cell survival *in vitro* by stimulating spleen cells from Dll1^−/−^: OT-II (Thy1.1^−^Thy1.2^+^) or control mice (Thy1.1^+^Thy1.2^+^) with OVA protein and observed a rapid decline of Dll1^−/−^: OT-II CD4^+^ T cells (Fig. 2b). T cell recall responses 30 days after immunization of OVA in Dll1^−/−^ mice were lower than that of control mice (Supplementary Figure 2b), which also supports there being a defect in activated CD4^+^ T cell survival in Dll1^−/−^ mice.

In order to evaluate the specificity of Dll1, we co-transferred T cells from Jagged1^−/−^ mice crossed with OT-II TCR transgenic (Jagged1^−/−^: OT-II) mice (Thy1.1^−^Thy1.2^+^) and Jagged1^+/+^: OT-II mice (Thy1.1^+^Thy1.2^+^) into C57BL/6 mice (Thy1.1^+^Thy1.2^−^) that were subsequently immunized with OVA protein emulsified in CFA. However, the relative cell number of Jagged1^−/−^: OT-II mice did not change 22 days after immunization (Fig. 2c). These findings suggest that Dll1, but not Jagged1, on CD4^+^ T cells is required for the survival of activated/memory CD4^+^ T cells.

### Induction of EAE is suppressed in Dll1^−/−^ but not Jagged1^−/−^ mice

**Figure 3:**
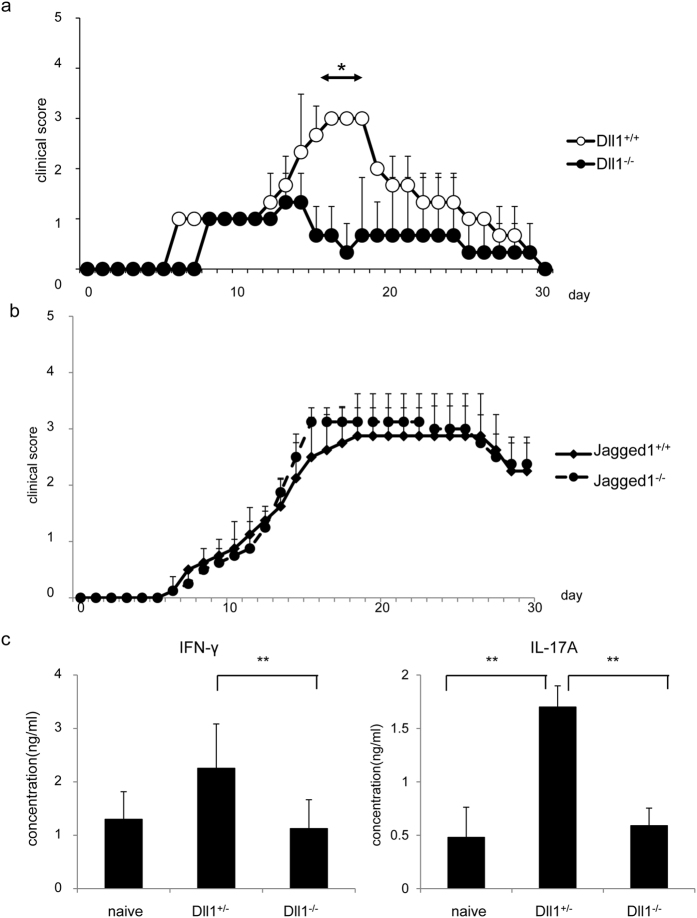
(**a**) Dll1^−/−^ or Dll1^+/+^ mice (n = 7) were immunized with MOG emulsified in CFA and given pertussis toxin on days 0 and 2. EAE clinical scores were measured on the indicated days. The data shown are mean ± S.D. *Indicates statistical difference (*p* < 0.05). (**b**) Jagged1^−/−^ or Jagged1^+/+^ mice (n = 7) were immunized with MOG emulsified in CFA and given pertussis toxin on days 0 and 2. EAE clinical scores were measured on the indicated days. The data shown are mean ± S.D. (**c**) T cells in draining lymph nodes 37 days after MOG immunization (n = 5) as shown in (**a**) were stimulated with MOG (20 μg/ml) for three days and the concentrations of IFN-γ and IL-17A were measured by ELISA. The data are shown as mean ± S.D. **Indicates statistical difference (*p* < 0.01). The data in these figures are representative of three independent experiments.

In order to assess whether Dll1 is important for the survival of activated/memory CD4^+^ T cells in pathological model, we evaluated the roles of Dll1 by experimental autoimmune encephalitis (EAE) model. Dll1^−/−^ and control mice were injected with MOG emulsified in CFA to induce EAE. The clinical scores up to 14 days after MOG immunization were comparable between the two groups. However, the clinical scores were lower in Dll1^−/−^ compared to Dll1^+/+^ mice after day 14 (Fig. 3a). We did not observe a difference of EAE induction in Jagged1^−/−^ mice (Fig. 3b). As Th1 and Th17 are crucial for the induction of EAE, T cells from Dll1^−/−^ and control mice 37 days after MOG immunization were cultured with MOG peptide for 3 days. The concentration of IFN-γ and IL-17A was much less in Dll1^−/−^ cultures than in control cultures (Fig. 3c). Fewer IFN-γ- and IL-17A-producing cells in Dll1^−/−^ but not Jagged1^−/−^ mice were also observed by flow cytometry 35 days after MOG immunization (Supplementary Figure 3). These data suggest that *Dll1* deficiency in CD4^+^ T cells suppresses EAE induction by inhibiting Th1 and Th17 survival.

### Unimpaired control of Leishmania major in Dll1^−/−^ mice

**Figure 4:**
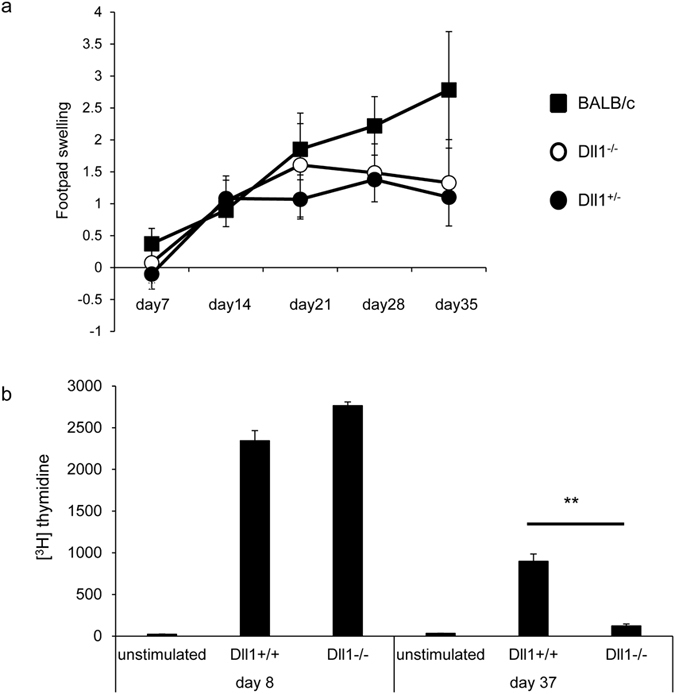
Mice were inoculated with 5 × 10^6^ metacyclic phase *L. major* in the left hind footpad (n = 8) and (**a**) the inoculated footpad thickness was measured weekly. (**b**) Purified CD4^+^ T cells 8 or 24 days after infection were stimulated with *L. major-*derived antigens for 3 days and [^3^H] thymidine incorporation during the final 6 hours was measured. Error bars represent mean ± S.D. **Indicates *p* < 0.01. The data are representative of two independent experiments.

To further evaluate the roles of Dll1 in the functional differentiation of CD4^+^ T cells *in vivo*, we infected Dll1^−/−^ mice with *Leishmania major (L. major*). The control of infection was evaluated by measuring footpad swelling. Dll1^−/−^ mice were resistant to *L. major* infection similar to control Dll^+/+^ mice (Fig. 4a). We continued to observe similar resistance in both mouse strains 50 days after infection. Lymph node cells were stimulated with *L. major*-derived antigens 8 or 37 days after infection and cell proliferation was evaluated (Fig. 4b). Proliferation was comparable at 8 days after infection, whereas the proliferation was less in Dll1^−/−^ than control mice 37 days after infection. These data demonstrate that initial T cell proliferation is unaffected by *Dll1* deficiency, which could confer protective immunity against *L. major*; however, activated cells do not survive in *Dll1*-deficient mice.

### Absence of Dll1 on T cells does not affect Notch expression and signaling

**Figure 5:**
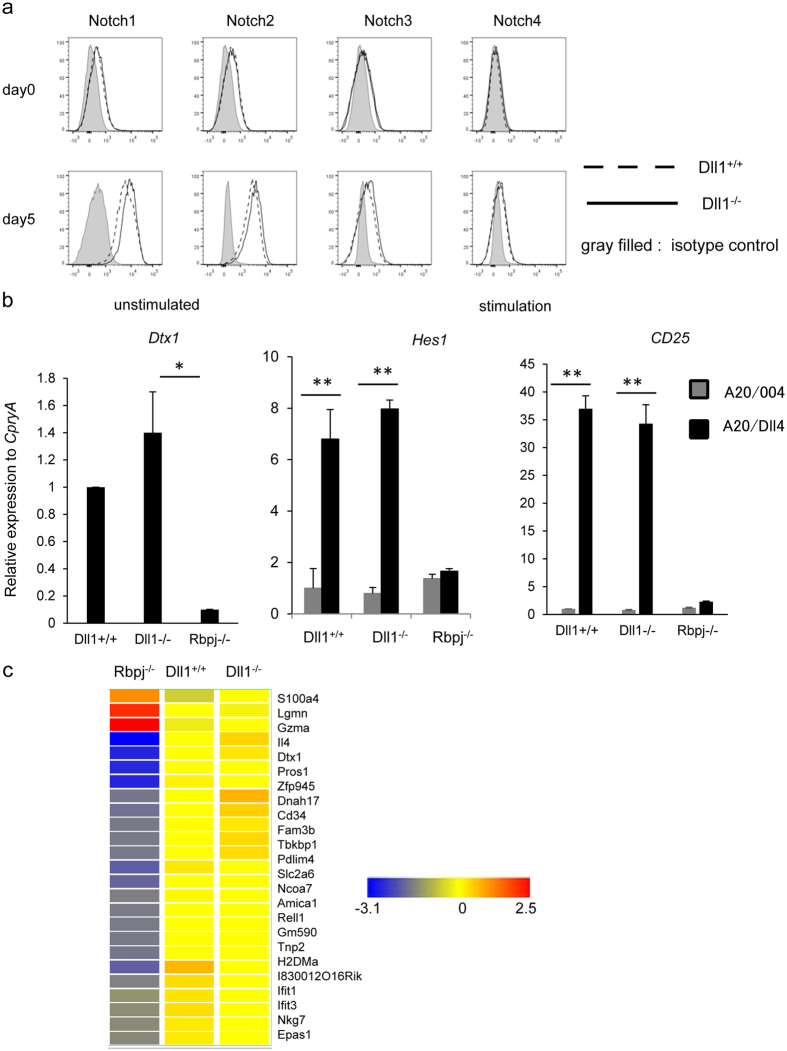
(**a**) The expression of Notch1, Notch2, Notch3 and Notch4 on naïve CD4^+^ T cells from Dll1^−/−^ (solid line) or Dll1^+/+^ (dotted line) mice or CD4^+^ T cells stimulated with anti-CD3 mAb for 2 days from Dll1^−/−^ or Dll1^+/+^ mice were evaluated by flow cytometry. Cells stained with isotype IgG were used as negative controls (shadow). The data in this figure are representative of three independent experiments. (**b**) The expression of *Dtx1, Hes1, Cd25* in naïve CD4^+^ T cells from Dll1^−/−^ or Dll1^+/+^ mice (n = 5) or CD4^+^ T cells stimulated with anti-CD3 mAb for ten days from Dll1^−/−^ or Dll1^+/+^ mice were evaluated by real-time PCR. The data shown are mean ± S.D. * or **Indicates statistical difference (*p* < 0.05, *p* < 0.01, respectively). (**c**) Total spleen cells from Rbpj^−/−^, Dll1^−/−^ or Dll1^+/+^ mice were stimulated with anit-CD3 mAb for three days and CD4^+^ T cells were sorted. The genes that are differentially expressed (more than three-fold higher or lower) between CD4^+^ T cells from Rbpj^−/−^ and Dll1^+/+^ mice are shown. The color changes indicate the Z-score. The data in these figures are representative of three independent experiments.

We reported previously that the deficiency of Notch signaling affected the survival of memory CD4^+^ T cells^22^. Therefore, we determined if the absence of Dll1 affects the expression of the different Notch receptors. The expression of Notch1, Notch2, Notch3, and Notch4 was comparable between CD4^+^ T cells from Dll1^−/−^: OT-II and Dll1^+/+^: OT-II mice before and after stimulation of CD4^+^ T cells with OVA peptide for 3 days (Fig. 5a). To further delineate if Dll1 deficiency in CD4^+^ T cells affects Notch signaling, we evaluated the Notch target genes *Dtx1, Hes1*, and *Cd25* in naïve and activated CD4^+^ T cells from Dll1^−/−^, Dll1^+/+^ or *Rbpj*^flox/flox^ mice crossed with CD4-*Cre* transgenic (Rbpj^−/−^) mice stimulated with anti-CD3 and anti-CD28 mAbs with the support of A20 cells or A20 cells overexpressing Dll4 (A20-Dll4) to overstimulate Notch for 10 days. The expression of *Dtx1* was less in naive CD4^+^ T cells from Rbpj^−/−^ mice than those from control mice, indicating that *Dtx1* transcription depends on Rbpj as previously reported. However, the expression of *Dtx1* in naive CD4^+^ T cells from Dll1^−/−^ mice was equivalent to that of control cells (Fig. 5b). The expression of *Hes1* and *Cd25* was upregulated in CD4^+^ T cells from control mice following stimulation with A20-Dll4, whereas upregulation was not observed in CD4^+^ T cells from Rbpj^−/−^ mice. A similar upregulation of *Hes1* and *Cd25* was detected in CD4^+^ T cells from Dll1^−/−^ mice. The level of cell-surface CD25 on CD4^+^ T cells from Dll1^−/−^ mice was equivalent to that of control cells (Supplementary Figure 4). We also compared the mRNA expression of genes that are reduced or upregulated in activated CD4^+^ T cells from Rbpj^−/−^ mice compared with control cells obtained from DNA microarray analysis. However, the expression of those genes was not altered in *Dll1*-deficient activated CD4^+^ T cells (Fig. 5c). We also evaluated glucose uptake by activated CD4^+^ T cells, because Rbpj-deficient activated CD4^+^ T cells have a defect in glucose uptake^12^. However, we did not observe impaired glucose uptake by Dll1-deficient CD4^+^ T cells (Supplementary Figure 4). Taken together, these data suggest that *Dll1* deficiency in CD4^+^ T cells does not affect the expression of Notch and Rbpj-mediated Notch signaling.

### The intracellular domain of Dll1 rescues T cell survival in Dll1^−/−^ mice

**Figure 6:**
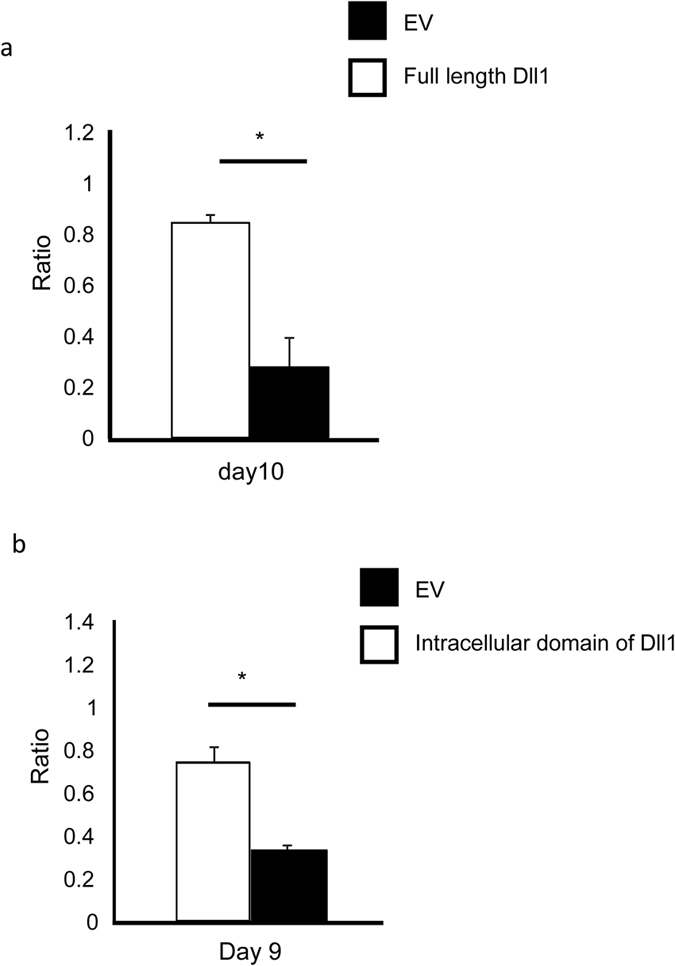
Spleen cells from Dll1^−/−^ (Thy1.1^−^Thy1.2^+^) or Dll1^+/+^ mice (Thy1.1^+^Thy1.2^+^) were stimulated with anti-CD3 mAb and a retrovirus carrying the full-length or intracellular domain of Dll1 or control retrovirus was infected twice after which GFP^+^CD4^+^ T cells were purified. (**a**) Retrovirus carrying the full-length of Dll1 infected CD4^+^ T cells from Dll1^−/−^ mice together with control vector-infected CD4^+^ T cells from Dll1^+/+^ mice were co-transferred into the same unirradiated recipient mice (Thy1.1^+^Thy1.2^−^, n = 12). (**b**) Retrovirus carrying the intracellular domain of Dll1 infected CD4^+^ T cells from Dll1^−/−^ mice together with control vector-infected CD4^+^ T cells from Dll1^+/+^ mice were co-transferred into the same unirradiated recipient mice (Thy1.1^+^Thy1.2^−^, n = 12). The ratios of T cells were compared 9 or 10 days after transfer. The data are shown as the ratio (black) of retrovirus carrying the full-length (**a**) or intracellular domain (**b**) of Dll1-infected CD4^+^ T cells from Dll1^−/−^ mice/control retrovirus-infected CD4^+^ T cells from Dll1^+/+^ mice. As the control, control retrovirus-infected CD4^+^ T cells from Dll1^−/−^ mice together with control vector-infected CD4^+^ T cells from Dll1^+/+^ mice were transferred into the same unirradiated recipient mice (Thy1.1^+^Thy1.2^−^, n = 12). The ratio (white) of control retrovirus-infected CD4^+^ T cells from Dll1^−/−^ mice/control retrovirus-infected CD4^+^ T cells from Dll1^+/+^ mice is shown. The day when cells were transferred is set as day 0. The data shown are mean ± S.D. *indicates statistical difference (*p* < 0.05). The data in these figures are representative of four independent experiments.

In order to investigate if Dll1 really transduces a survival signal in activated CD4^+^ T cells, we transduced a retrovirus encoding the full-length or intracellular domain of Dll1 or control virus into activated CD4^+^ T cells from Dll1^−/−^ or Dll1^+/+^ mice, which were subsequently transferred into unirradiated C57BL/6 mice. The relative percentage of T cells from Dll1^−/−^ versus those from Dll1^+/+^ mice was compared on days 4 and 13 or 14, and the fold increase at day 13 or 14 was calculated, considering day 4 to be one. Transducing full-length Dll1 rescued cell survival of Dll1^−/−^ CD4^+^ T cells compared with control virus-infected cells (Fig. 6a). Transducing only the intracellular domain of Dll1 partially rescued cell survival of Dll1^−/−^ CD4^+^ T cells (Fig. 6b). These data suggest that direct signaling through Dll1 through cleavage of Dll1 is, at least partially, necessary for the survival of activated CD4^+^ T cells.

## Discussion

Notch regulates a variety of aspects of T cell differentiation including helper T cell differentiation and cytotoxic T cell differentiation^5^,^6^,^7^,^8^,^9^,^10^,^11^. Furthermore, our group recently reported that Notch signaling is essential for the maintenance of memory CD4^+^ T cells, at least partly due to interactions with Dll1^22^. Although Notch ligands interact in trans with Notch receptors expressed on adjacent cells, previous studies have revealed the cis-inhibition of Notch signaling by Notch ligands on the same cells^15^. However, it remains unclear whether this cis-regulation is involved in Notch signaling in T cells. In this report we showed that Dll1 on T cells is required for the maintenance of activated CD4^+^ T cells without affecting Notch signaling on the same cells. This novel mechanism by which Dll1 transmits survival signals in activated CD4^+^ T cells provides an important insight into not only the regulation of maintenance of activated CD4^+^ T cells but also the physiological roles of Dll1.

Previous studies reported that Notch signaling is inhibited by the presence of excess ligand on the same cell^15^. However, in our present studies, the deletion of *Dll1* in CD4^+^ T cells did not affect Rbpj-mediated Notch target genes. Furthermore, deletion of *Dll1* in CD4^+^ T cells did not alter the expression of Notch receptors. These findings suggest that the deletion of *Dll1* in CD4^+^ T cells does not affect Notch signaling in these cells through cis interactions. Nevertheless, one might argue that the impaired survival of activated CD4^+^ T cells^22^ from T cell-specific Rbpj-deficient mice being similar to those of Dll1^−/−^ mice *in vivo* is attributable to the requirement of Dll1 expressed on CD4^+^ T cells, which interacts with Notch on adjacent CD4^+^ T cells. Similarly, the interaction of Dll1 and unknown Dll1 receptors might be crucial for T cell survival. However these possibilities are unlikely because we transferred CD4^+^ T cells from Dll1^−/−^ mice into recipient mice where large numbers of recipient T cells able to express Dll1 are present and presumably interacting with CD4^+^ T cells from Dll1^−/−^ mice. Furthermore, it is possible that Dll1on activated CD4^+^ T cells provides a signal to antigen-presenting cells that is required for supporting T cell survival, and that neighboring T cells cannot receive the survival signal from antigen-presenting cells. Although we cannot completely rule out this possibility, we demonstrated that transduction of the intracellular domain of Dll1 in *Dll1*-deficient cells could partially rescue the survival of activated CD4^+^ T cells, indicating that Dll1 can, at least partially, transmit a survival signal into activated CD4^+^ T cells.

Several studies have revealed that the cleaved intracellular Notch ligands translocate into the nucleus where they regulate gene expression^20^. For instance, the soluble Jagged intracellular domain can activate gene expression via AP1^20^. The intracelllular domain of Dll1 binds specifically to Smad2, Smad3 and Smad4, which are involved in TGF-β**/**activin signaling^21^. However, these studies have not assessed physiological roles for Notch ligands-mediated signaling by using loss-of-functions systems or *in vivo* experiments. Our present study is the first to demonstrate a physiological role for Dll1-mediated signal transmission *in vivo*. Furthermore, our data suggest that Dll1-mediated signals in non-T cells would also likely have some physiological impact. Therefore, in general, we should consider Dll1-expressing cells as not only Notch signal sending cells but also Dll1 signal receiving cells when analyzing Notch signaling-mediated cellular interactions.

Dll1^−/−^ mice had lower EAE scores than control mice, and Dll1^−/−^ mice were able to mount protective immune responses against *L. major*. Th1 differentiation was not affected early after either MOG immunization, *L. major* infection and *in vitro* Th1-skewing experiments. However, the proliferation of CD4^+^ T cells was less in Dll1^−/−^ mice late after MOG immunization and *L. major* infection. The reason why Dll1^−/−^ mice could control *L. major* but exhibit lower EAE scores is that long-term persistence of functional CD4^+^ T cells is needed for EAE pathology but not for control of *L. major*. In any case, *Dll1*-deficient CD4^+^ T cells are able to mount initial effector functions in CD4^+^ T cells *in vivo* but the long term maintenance of effector cells including Th1 and Th17 cells would be impaired by *Dll1* deficiency.

In conclusion, we demonstrated that the deletion of *Dll1* in CD4^+^ T cells disturbs the survival of activated CD4^+^ T cells. The impairment of CD4^+^ T cell survival is attributable to the lack of signaling through Dll1. Our data reveal previously undefined Dll1-mediated regulation of T cell survival and identify Dll1 as a novel regulator for maintaining adaptive T cell immune responses.

## Materials and Methods

### Mice

Female C57BL/6 mice were purchased from Japan SLC (Hamamatsu, Japan). OT-II T cell receptor transgenic mice were purchased from Taconic (NY, USA). *Rbpj*^flox/flox^^10^, *Dll1*^flox/flox^^23^, *Jagged1*^flox/flox^^24^ and CD4-*Cre* transgenic^10^ mice were previously reported. All mice were backcrossed onto C57BL/6 at least 10 times. Dll1^f/f^ mice crossed with CD4-*Cre* transgenic mice or control Dll1^+/+^ mice crossed with CD4-*Cre* transgenic mice were obtained by crossing Dll1^f/+^ mice crossed with Dll1^f/+^ mice with CD4-*Cre* transgenic mice. All mice were maintained under specific pathogen-free conditions in the animal research center of Tokushima University and all experiments were performed in accordance with our institution’s guidelines for animal care and use. All experimental protocols were approved by the animal research committee of Tokushima University.

### Flow cytometry

Cells from the thymus, spleen or lymph nodes were resuspended in staining buffer at a density of 2 × 10^6^ cells/ml. The cells were incubated with anti-*Fcγ*RII/III monoclonal antibody (mAb) (2.4G2), followed by antibodies against CD4 (RM4-5, BD Pharmingen), CD8 (53–6.7, BD Pharmingen), B220 (RA3-6B2, BioLegend), Thy1.1 (53–2.1, BioLegend), Thy1.2 (OX-7, BioLegend), CD44 (IM7, BioLegend), CD62L (MEL-14, BioLegend), Dll1 (HMD1-5, eBiosciences), Dll4 (HMD4-1, eBiosciences), Jagged1 (HMJ1-29, eBiosciences) or Jagged2 (HMJ2-1, eBiosciences). After gating out cells that were positive for 7-AAD, excluding the doublets, the fluorescence intensity of 10^5^ cells was measured with a FACS Canto II flow cytometer (BD Biosciences, CA) and analyzed with FACSDiva (BD Biosciences) or FlowJo (Tree Star) software programs. For intracellular staining, cells were fixed with an intracellular staining kit (72–5775, eBioscience), permeabilized and stained with Foxp3 (3G3, Tonbo Biosciences) antibody.

### ELISA

The concentrations of IL-4, IL-17A and IFN-γ were measured by Ready-Set-Go ELISA kits (eBioscience) according to the manufacturer’s protocol.

### Cell culture

Lymph node cells were cultured for 3 days in RPMI 1640 supplemented with 10% (v/v) heat-inactivated FCS, 5 mM l-glutamine, 50 μM 2-ME, and antibiotics (Penicillin-Streptomycin) in the presence of 20 μg/ml MOG_35–55_ peptide under Th1 (IL-12 10 μg/ml and anti-IL-4 10 μg/ml), Th2 (IL-4; 30 ng/ml and anti-IFN-γ; 10 μg/ml) or Th17-polarizing (IL-6; 10 ng/ml, TGF-β1; 1 ng/ml, anti-IFN-γ; 10 μg/ml and anti-IL-4; 10 μg/ml) conditions. T cells purified by CD4 microbeads and LS columns (Miltenyi Biotec) were stimulated with plate-bound anti-CD3 and anti-CD28 for 24 hours to perform ELISA assay. GeneJuice (Merck Millipore) was used to generate retroviruses by transfecting each vector encoding GFP into Plat-E cells (provided by Dr. T. Kitamura)^25^. T cells purified by CD4 microbeads and LS columns (Miltenyi Biotec) were stimulated with plate-bound anti-CD3 and anti-CD28 for 24 hours. After the addition of supernatant containing retroviruses carrying *Dll1* or the intracellular domain of *Dll1*, cells were centrifuged at 2600 rpm for 1 hour two times at 8-hour intervals.

### Real-time PCR

Total RNA was isolated (ReliaPrep RNA Cell Miniprep System, Promega) and cDNA was synthesized (0.1 μg of RNA). Relative expression was calculated using the comparative threshold cycle method. qRT-PCR analyses were performed by using StepOnePlus (Applied Biosystems) with primers; *Bcl2l11* forward 5′-CACTGGTTGCTGGCTTTGCTG-3′, reverse 5′-TCCTTGCTCCTGAAATGACCTGG-3′, *Cflar* forward 5′-TCCAGAATGGGCGAAGTAAAGAGC-3′, reverse 5′-AGTCTCTTCACGGATGTGCGGAG-3′, *Bcl2l1* forward 5′-AACATCCCAGCTTCACATAACCCC-3′, reverse 5′-GCGACCCCAGTTTACTCCATCC-3′, *Bid* forward 5′-AATCATCCACAACATTGCCAGA-3′, reverse 5′-GCCTTGTCGTTCTCCATGTCT, *Dll1* forward 5′-TGCTCCGAAACAAGAGTGTG-3′, reverse 5′-CAGGCAGAGCAGGTGATACA-3′, *Jagged1* forward 5′-CTTGGGTCTGTTGCTTGGTG-3′, reverse 5′-GTGTTGGCTCCGTGTTTCTC-3′, *Cd25* forward 5′-CCTGGCAACACAGATGGAG-3′, reverse 5′-AGCCGTTAGGTGAATGCTTG-3′, *CypA* forward 5′-AGCATACAGGTCCTGGCATC-3′, reverse 5′-TTCACCTTCCCAAAGACCAC-3′. The mRNA level of *CypA* was used as an internal control.

### EAE

Mice were subcutaneously immunized with 200 μg MOG_35–55_ peptide emulsified in CFA containing 500 μg heat-killed *Mycobacterium tuberculosis* (H37RA; Difco Laboratories). Mice were injected intraperitoneally with 200 ng pertussis toxin on day 0 and 2 days after MOG immunization. Clinical signs were assessed daily: 0, no symptoms of disease; 1, loss of tail tonicity; 2, hindlimb weakness/impaired gait; 3, partial hindlimb paralysis; 4, complete hindlimb paralysis; 5, forelimb paralysis or moribund. Lymph node cells at day 37 were cultured for 3 days in RPMI 1640 supplemented with 10% (v/v) heat-inactivated FCS, 5 mM l-glutamine, 50 μM 2-ME, and antibiotics in the presence of 20 μg/ml MOG_35–55_ peptide.

### Leishmania major infection

Mice at the age of 8 weeks were subcutaneously inoculated with 5 × 10^6^ metacyclic phase *Leishmania major* (MHOM/SU/73/5ASKH). The infection was monitored by weekly measurements of the infected footpad swelling. CD4^+^ T cells from the draining lymph nodes of infected mice purified with CD4 microbeads (Miltenyl Biotech) 8 or 37 days after infection were stimulated with Leishmania major-derived antigens in the presence of irradiated spleen cells from C57BL/6 mice for 3 days and [^3^H]-thymidine incorporation during the final 6 hours was measured by a liquid scintillation counter.

### T cell survival assay

Purified CD4^+^ T cells (5 × 10^6^) with CD4 microbeads ((Miltenyi Biotec) were transferred into unirradiated C57BL/6 mice. In some experiments, mice were immunized with OVA protein (50 μg/mouse) emulsified in complete Freund’s adjuvant (SLBD0737, Sigma). In some experiments, T cells were stimulated with anti-CD3 mAb (1 μg/ml) and infected two times with retrovirus, as previously reported^10^. The infection efficiency was similar (30~40%) between control virus and Dll1-carrying virus (data not shown). GFP-positive cells were purified four days after stimulation by cell sorting (FACSAria III, BD Biosciences) for transfer into recipient mice. For *in vitro* T cell survival assay, purified CD4^+^ T cells by CD4 microbeads (Miltenyi Biotec) from Dll1^−/−^: OT-II (Thy1.1^−^Thy1.2^+^) or Dll1^+/+^: OT-II (Thy1.1^+^ Thy1.2^+^) mice were stimulated with irradiated spleen cells from C57BL/6 mice (Thy1.1^+^ Thy1.2^−^) and OVA protein (50 μg/ml). The ratio of Thy1.1^+^ and Thy1.1^−^ cells gated on Thy1.2^+^ cells were evaluated.

### Microarray

Total RNA was isolated using the Relia^TM^ RNA Cell Miniprep System (Promega) and RNA quality was assessed by analysis with an Agilent 2100 BioAnalyzer. Thirty ng of RNA was used for the RNA probe. Probe preparation and microarray analyses were performed on Whole Mouse Genome OligoDNA microarray kit ver2.0 4 × 44K (Agilent Technologies). The resulting data were normalized using GeneSpring (Agilent Technologies) software. Genes expressed ≥3.0-fold up or down (*p* < 0.05) between groups were considered to be differentially expressed.

### Statistical analysis

Statistical significance was evaluated by an unpaired, two-tailed *t* test or parametric Dunnett’s test. A *p* value of <0.05 was considered significant.

## Additional Information

**How to cite this article**: Furukawa, T. *et al*. Transmission of survival signals through Delta-like 1 on activated CD4^+^ T cells. *Sci. Rep.* **6**, 33692; doi: 10.1038/srep33692 (2016).

## Supplementary Material

Supplementary Information
