# Supplementary material for: Transmission of survival signals through Delta-like 1 on activated CD4+ T cells
Source: Sci Rep. 2016 Sep 23;6:33692. doi: 10.1038/srep33692 (PMC5034251; doi:10.1038/srep33692)
Supplement: Supplementary Information [file srep33692-s1.pdf]

Transmission of survival signals through Delta-like 1 on activated $CD4^+$ T cells

Takahiro Furukawa, Chieko Ishifune, Shin-ichi Tsukumo, Katsuto Hozumi, Yoichi Maekawa, Naoko Matsui, Ryuji Kaji, Koji Yasutomo

Supplementary Figure 1. Furukawa, et al.

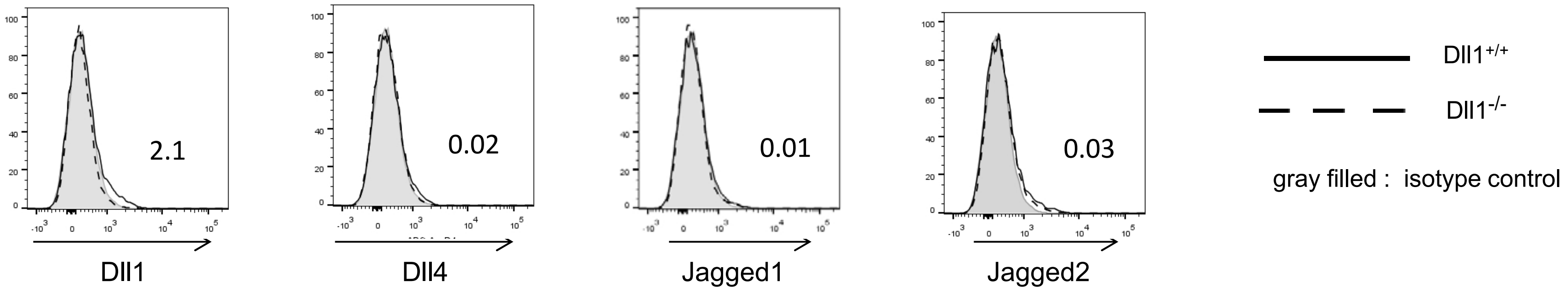

Figure 1. Expression of Notch ligands in activated CD4⁺ T cells

Spleen T cells from Dll1$^{+/+}$ (solid line) or Dll1$^{-/-}$ mice (dotted line) (n=5) were stimulated with anti-CD3 mAb for 5 days. Cells were stained with antibodies to CD4 and Dll1, Dll4, Jagged1 or Jagged2, and then analyzed Dll1, Dll4, Jagged1 or Jagged2 expression on CD4^{+} cells by flow cytometry. Cells stained with isotype control antibodies were used as a negative control (shadow). The number in the figures are percentage of positive cells. The data in this figure are representative of three independent experiments.

Supplementary Figure 2. Furukawa, et al.

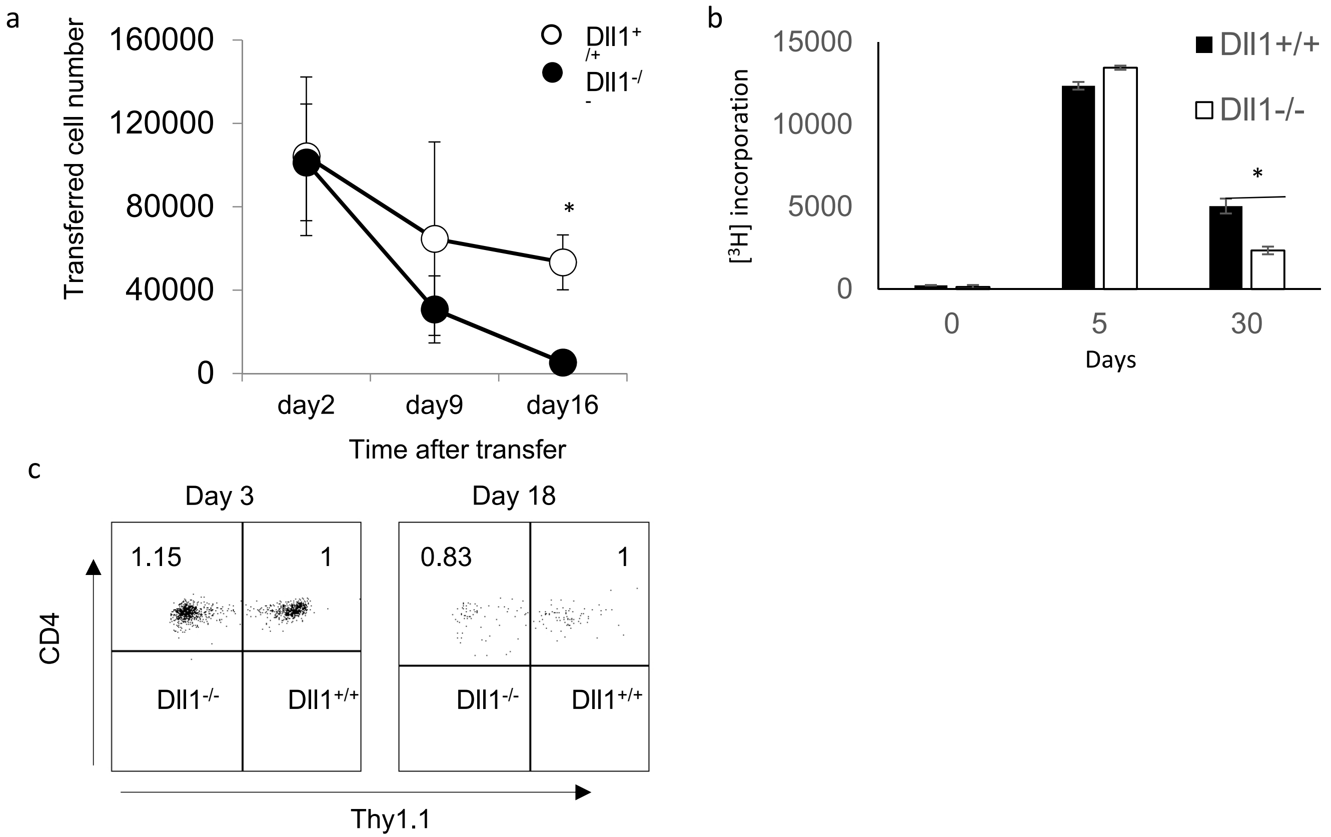

Figure 2. Rapid loss of Dll1-negative CD4$^+$ T cells

(a)Identical numbers of T cells from Dll1$^{-/-}$: OT-II (Thy1.2$^+$) or Dll1$^{+/+}$: OT-II (Thy1.1$^+$) mice were transferred into unirradiated C57BL/6 (Thy1.1$^+$Thy1.2$^+$) mice (n=6) that were subsequently immunized with OVA in CFA. The actual numbers of Thy1.1$^-$Thy1.2$^+$ or Thy1.1$^+$Thy1.2$^-$ cells 2 or 16 days after immunization were counted and shown as mean $\pm$ S.D. * indicates a statistical difference at $P<0.05$. The data in this figure are representative of three independent experiments. (b) Dll1$^{-/-}$ (open) or Dll1$^{+/+}$ (closed) mice (n=5) were immunized with OVA protein (100 µg) emulsified in CFA and total lymph nodes cells were harvested 5 or 30 days later and stimulated with OVA protein for 3 days. T cell proliferation was evaluated by [^{3}H]-thymidine incorporation during the final 6 hours. The data are shown as mean $\pm$ S.D. * indicates a statistical difference at $P<0.05$. The data in this figure are representative of three independent experiments. (c) CD4$^+$ T cells purified by microbeads from Dll1$^{+/+}$ (Thy 1.1$^+$Thy1.2$^-$) or Dll1$^{-/-}$ (Thy1.1$^-$Thy1.2$^+$) mice were transferred into same unirradiated recipient mice (Thy1.1$^+$Thy1.2$^+$) (n=5). The frequency of donor cells was evaluated 3 or 18 days after transfer by flow cytometry. The data in this figure are representative of three independent experiments.

Supplementary Figure 3. Furukawa, et al.

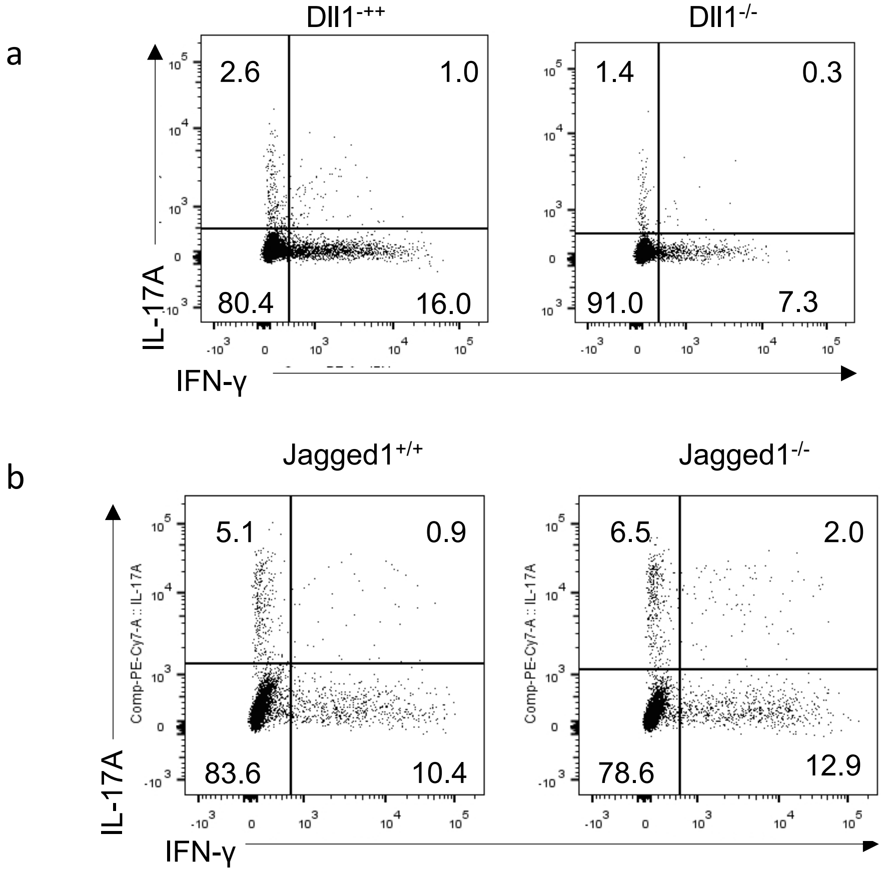

Figure 3. Reduced IL-17A and IFN-γ in CD4$^+$ T cells of Dll1$^{-/-}$ mice immunized by MOG peptide

Lymph node cells (a) Dll1$^{-/-}$, (b) Jagged1$^{-/-}$ or control mice (n=5) were harvested 35 days after MOG immunization and were cultured for 3 days in the presence of 20 µg/ml $MOG_{35–55}$ peptide. Cells were stimulated with PMA and ionomycin for 5 hours in the presence of monensin, stained with anti-CD4, IFN-γ and IL-17A antibodies and analyzed by flow cytometry. The data in this figure are representative of three independent experiments.

Supplementary Figure 4. Furukawa, et al.

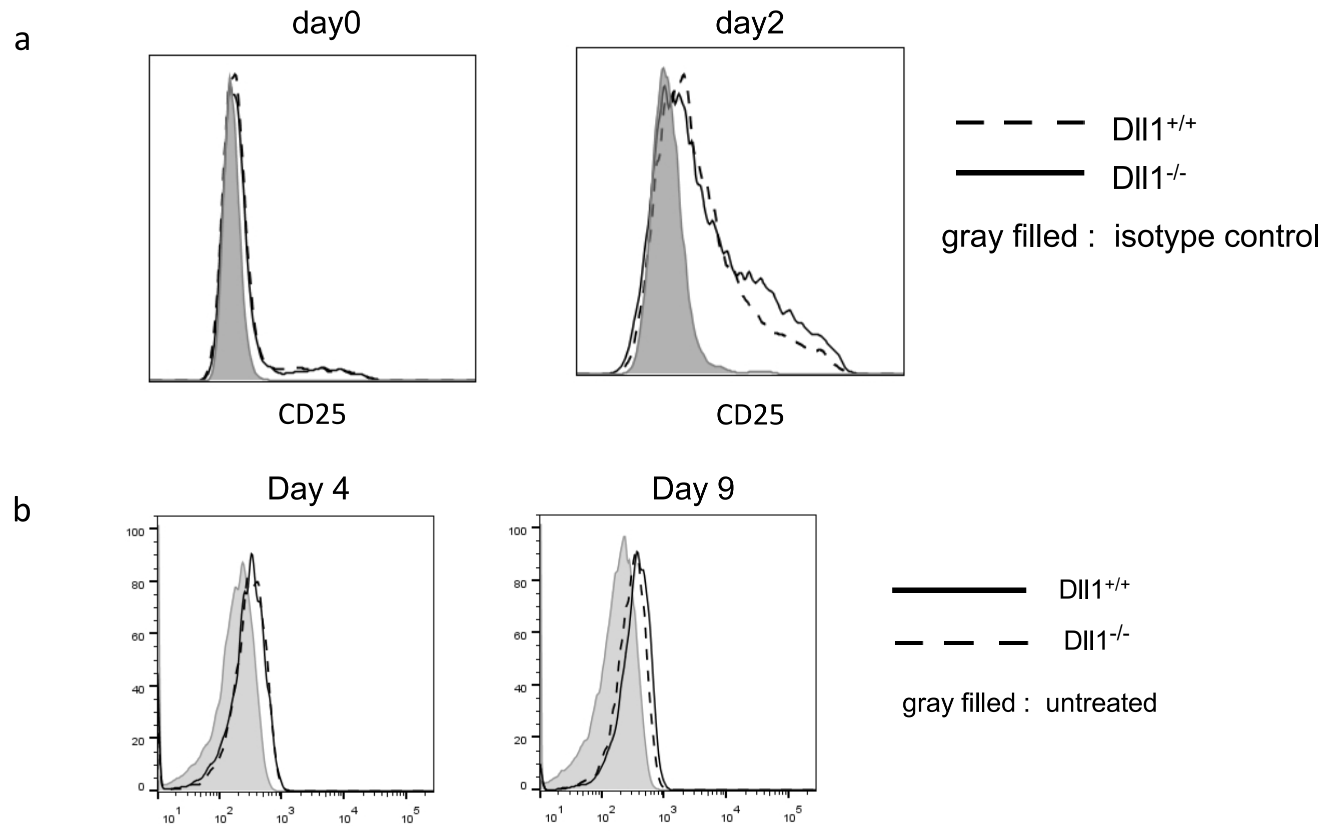

Figure 4. Unimpaired CD25 expression and glucose uptake in $CD4^+$ T cells in $Dll1^{-/-}$ mice

(a)Spleen T cells from $Dll1^{+/+}$ (dotted line) or $Dll1^{-/-}$ (solid line) mice were stimulated with anti-CD3 mAb (1 µg/ml) for 2 days, and CD25 expression on $CD4^+$ T cells was evaluated by flow cytometry. As the negative control, cells stained with an isotype control antibody were used (gray shadow). The data in this figure are representative of three independent experiments. (b) Purified $CD4^+$ T cells (2 x 10^6) from $Dll1^{+/+}$: OT-II (solid line) or $Dll1^{-/-}$: OT-II mice (dotted line) were transferred into C57BL/6 (Thy1.1) mice (n=8), that were then immunized with OVA protein emulsified in CFA. Four or 9 days after immunization, FITC-conjugated 2-(N-(7-nitrobenz-2-oxa-1,3-diaxol-4-yl) amino)-2-deoxyglucose (2-NBDG) (2 mM) (Molecular Probes) was injected intravenously into mice. One hour later, spleen and draining lymph node cells were collected and stained with antibodies to CD4, CD44, Thy1.1 and Thy1.2 for flow cytometry. As the negative control, untreated cells were used (gray shadow). The data are shown gated on $CD4^+CD44^+Thy1.2^+$ cells. The data in this figure are representative of three independent experiments.
